# Assessing trauma in a transcultural context: challenges in mental health care with immigrants and refugees

**DOI:** 10.1186/s40985-018-0102-y

**Published:** 2018-08-22

**Authors:** Lloy Wylie, Rita Van Meyel, Heather Harder, Javeed Sukhera, Cathy Luc, Hooman Ganjavi, Mohamad Elfakhani, Nancy Wardrop

**Affiliations:** 1 Department of Psychiatry, Schulich School of Medicine and Dentistry, Western University, London, Ontario Canada; 2 London Health Sciences Centre, London, Ontario Canada; 3 Schulich Interfaculty Program in Public Health, Western University, London, Ontario Canada

**Keywords:** Immigrants, Refugees, Mental health, Assessments, Trauma, Transcultural, Qualitative methods

## Abstract

The growing numbers of refugees and immigrants from conflict-prone areas settling throughout the world bring several challenges for those working in the mental health care system. Immigrants and refugees of all ages arrive with complex and nuanced mental health histories of war, torture, and strenuous migration journeys. Many of the challenges of addressing the health care needs for this growing population of immigrants and refugees are often unfamiliar, and thus practices to address these challenges are not yet routine for care providers and health care organizations. In particular, complex trauma can make mental health assessments difficult for health care organizations or care providers with limited experience and training in transcultural or trauma-informed care. Using a transcultural approach can improve assessment and screening processes, leading to more effective and high-quality care for immigrant and refugee families experiencing mental health disorders.

This paper presents findings from an assessment of current mental health services focusing on current practices and experiences with immigrant and refugee patients and families. The difficulties in developing shared understandings about mental health can hinder the therapeutic process; therefore, it is imperative to ensure an effective assessment right from the beginning, yet there is limited use of existing cultural formulation tools from the DSM-IV or DSM-5. The paper outlines current practices, approaches, challenges, and recommendations shared by mental health care providers and program leaders in addressing the mental health care needs of immigrants and refugees. The results from this study demonstrate that there are many challenges and inconsistencies in providing transcultural, trauma-informed care. Respondents emphasized the need for a thorough yet flexible and adaptive approach that allows for an exploration of differences in cultural interpretations of mental health. Our study concluded that ensuring a mindful, reflexive, transcultural, and trauma-informed health care workforce, and a learning environment to support staff with education, resources, and tools will improve the health care experiences of immigrants and refugees in the mental health care system.

## Introduction

The growing numbers of refugees and immigrants from conflict-prone areas settling throughout the world bring several challenges for those working in the mental health care system. The high prevalence of stressful and traumatic experiences facing entire families lead to mental health issues that are different than what health care providers in countries not affected by war are accustomed to seeing [1, 2]. High numbers of immigrants and refugees of all ages have complex and multilayered mental health narratives, experiences of war, torture, and potentially traumatic migration journeys [3, 4]. As Vukčević et al. assert, “In order to ensure adequate psychological support [for refugees and asylum seekers], it is necessary to be aware of their traumatic experiences and the hardships they are dealing with” [2]. Mental health care providers need skills in trauma-informed care to meet the needs of people migrating from war-torn areas.

Assessment tools, such as the cultural formulation in the Diagnostic and Statistical Manual of Mental Disorders IV, or DSM-IV [5], provides an important foundation for the “evaluation of socio-cultural context of illness” [6], to assess this socio-cultural context of war and trauma. Despite the existence of practice guidelines for transcultural care, such as those outlined in the DSM-IV cultural formulation and in the DSM-5 [7], this assessment tool is underutilized in clinical settings [6, 8, 9] with care providers expressing concern with the time burden of such an assessment process [10]. This article discusses the challenges that mental health care providers working in London Ontario face with mental health assessments and care with immigrant and refugee patients. It identifies some of the barriers to using evidence-based models of care and provides recommendations from health care providers on how to support them to gain these practice-based skills.

## Background

A lack of transcultural care is only one of a range of access barriers in mental health care that lead to the underutilization of mental health services by immigrants and refugees [11], and these barriers exist both at the individual and system levels [12]. Barriers on the patient side include “a lack of knowledge of available services, difficulties with language and communications, and stigma associated with mental health problems and the use of services” [13]. Barriers from within the health care system include the limited transcultural knowledge, skills, and practices among care providers, as well as systemic time constraints that undermine the ability to carry out a fulsome sociocultural assessment. Complex trauma, such as that resulting from war, displacement and resettlement, can make mental health assessments difficult for health care organizations or care providers with limited experience and training in trauma-informed care, leading to misunderstandings [14, 15]. Using a transcultural approach, where practices are informed by this more thorough sociocultural assessment, can improve assessment and screening processes, leading to more effective and culturally appropriate care plans for immigrant and refugee families experiencing mental health disorders [16].

The recent diasporas of immigrants and refugees from war-torn areas relocating around the globe are changing demographics in profound ways. The Syrian conflict starting in 2011 has resulted in the world’s largest refugee crisis in the twenty-first century, resulting in 4.9 million Syrians registered as refugees by the end of 2016 [17]. From 2011 to 2016, a reported 29,945 Syrian refugees immigrated to Canada, with 1235 of these Syrian refugees migrating to London, Ontario, census metropolitan area (CMA). Immigrants born in India were the second largest group of recent arrivals to settle in London (CMA) with 1000 people. This influx influenced the 16% (47,955) to 20% (78,330) shift to London’s self-claimed visible minority population from 2011 to 2016 respectively. The largest increases are in the Arab and South Asian population, with an increase from 7715 to 13,735 (56% increase) and 6270 to 12,090 (51% increase) respectively. Between 2011 and 2016, 11,955 immigrants arrived in London, Ontario, resulting in a total 83,770 immigrants living in the city by the end of 2016 [18]. The people who live in London that neither speak English nor French increased from 4923 to 6320 from 2006 to 2016 [18, 19]. Patient profiles reflect the growth in the immigrant and non-English speakers in the city, including the increase in refugees of war. High levels of trauma and migration stresses among recently arrived war refugees [20] have profound significance in mental health care, where services generally are poorly equipped to meet the complex mental health needs of survivors of war and torture [11]. Many of the challenges of addressing the health care needs for this growing population of immigrants and refugees are therefore new and unfamiliar to care providers and health care organizations.

In the context of a global diaspora of war refugees, complex trauma poses several challenges to the provision of mental health services in many countries [16]. In a study on refugees and internally displaced people, Yeoman and Forman note that “PTSD may be an overly narrow characterization of traumatic stress across these different cultures” emphasizing instead experiences of “somatization, anxiety, and depression” [21]. Lewis-Fernandez have similarly noted the importance of understanding sociocultural contexts, which shape the way trauma is experienced and expressed [6]. Vukčević et al. reinforce this point, noting that “We should reconsider our familiar models of psychotherapy in order to accommodate the possibly different meanings of mental health, trauma, and support in the refugees’ cultural context” [2]. The difficulties in developing shared understandings about mental health can hinder the therapeutic process and influence assessment outcomes. Sundvall et al. emphasize that “there could be differences in the assessment of the patient based on the clinician’s assumptions and understanding of cultural factors” [14].

Such challenges highlight the importance of ensuring effective assessments are conducted at the first stages of an individual’s care journey. For health professionals who are ill equipped or inexperienced in providing care for culturally complex patients, modifying assessment processes in the context of complex structural and organizational processes are quite challenging. At the same time, the need to support immigrant and refugee families through the complexities of mental illness demands that innovative approaches and improved standards for transcultural and trauma-informed mental health care are put in place. This paper explores the challenges and recommendations to improve assessment protocols to meet the mental health needs of vulnerable immigrant and refugee populations with trauma histories.

Community forums were held to identify the challenges experienced by immigrants and refugees in mental health services in London, Ontario. These forums brought together people from across community and health care settings, including representation from immigrant settlement organizations, community services, educational institutions, community mental health groups, hospital representatives, and people with lived experience. An advisory committee was established with over 30 representatives from community stakeholders and organizations involved in immigrant and refugee services from both community and hospital mental health services, public health, social services, child welfare, settlement, education sectors, and additional members with lived experience. This committee regularly provided feedback on the research findings and practice recommendations for transcultural mental health care.

A range of gaps in access to culturally appropriate mental health services that meet the needs of immigrant and refugee communities in the London and Middlesex area of Southwestern Ontario were identified. Problems such as long waiting times, unavailability of interpreters, lack of trauma-informed care, and poor coordination between hospital and community-based services were some of the key issues that impeded the provision of effective, coordinated, and integrated mental health care. In 2013, a quality improvement process was initiated by an academic health sciences center in London, Ontario, to improve the mental health outcomes of immigrant and refugee children and families, through service changes in the hospital as well as enhancing collaboration between health and social service providers in community-based settings. To identify broader strategies for system improvement, exploring skills and gaps among health care providers was an important first step. This paper presents findings from a baseline assessment to gauge the current practices and experiences in providing mental health services to immigrant and refugee communities. The study captured a range of experiences among mental health care staff, aimed at understanding the current challenges and promising strategies of providing trauma-informed and culturally appropriate mental health care for immigrants and refugees, from assessments through treatment and recovery.

## Methods

A baseline assessment of mental health services for immigrants and refugees in London, Ontario, was completed in 2015. The study included semi-structured interviews and focus groups with health care providers and managers working within the continuum of mental health services both in the hospital system and hospital-affiliated community care settings. Respondents were asked about their experiences working with immigrant and refugee patients and families in mental health care. Questions included details around knowledge, skills, protocols, practices, education, and partnerships with community-based services. The purpose of the interviews was to develop a baseline of current attitudes, knowledge, and skills around culturally sensitive and competent practice within mental health and emergency care and to identify some of the main challenges that care providers were facing in transcultural mental health care contexts.

Approval for the study was received by the Health Sciences Research Ethics Board of Western University, and the ethics board of the Lawson Health Research Institute, ensuring adherence to the guidelines of the Tri-Agency Framework on Responsible Conduct of Research. Through a process of stratified purposeful sampling to assess experiences in providing transcultural mental health care, our respondents included mental health and emergency department physicians, psychiatrists, psychologists, residents, nurses, social workers, child and family therapists, employment and life skills counselors, occupational therapists, support staff, and organizational and program leaders within mental health. A total of 26 qualitative interviews and three focus groups were completed totaling 40 participants. The interviews examined both positive and negative experiences with immigrant and refugee patients; knowledge and training regarding protocols and services; and recommendations on ways to improve mental health services for immigrant and refugee patients.

Interviews and focus groups were recorded, transcribed verbatim, and coded in NVivo, using a grounded theory approach [22] to identify thematic nodes and sub-nodes based on the participant transcripts. There were 15 distinct nodes related to assessments, ranging from the type of assessment tools, to the challenges of the assessment process with this population. This paper presents and discusses the key findings from the themes around assessment practices, culminating in recommendations for improved practice based on standards such as the DSM cultural formulations, combined with local perspectives on strategies for advancing transcultural, trauma-informed mental health care.

## Results

The results from this study demonstrate that standards of practice for transcultural care are not in place across mental health services in London, Ontario, despite the existence of evidence-based recommendations in the DSM-5. This section outlines current practices, approaches, challenges, and recommendations shared by mental health care providers and program leaders in addressing the mental health care needs of immigrants and refugees.

### The inconsistency of current practices for transcultural assessment

Respondents shared that there was no common process guiding transcultural assessments in mental health. Although the DSM-5 provides a valuable framework for explaining cultural factors in the presentation of mental illness, there are financial, bureaucratic, ideological, and political barriers to making meaningful changes including implementing best practice guidelines across a large hospital. Health providers’ knowledge and practice skills in transcultural care vary widely. Patients also have varied pathways into mental health care, including the emergency department, direct admission, and referrals from doctors or community agencies; different assessments are carried out that provide different information. As one occupational therapist explained, regardless of how a patient accesses mental health services, background patient information is

Not always sent with the referral. I mean, if they come out of emerg [the Emergency Department] you don’t get everything. They’re going to focus on immediate crisis and what have you. Resources in the community might have their own information, but that’s not necessarily in a hospital chart. If the referral comes from there, hopefully there would be some appropriate information that would get sent about the client. That’s, I think, an area that can be really problematic. Often I get clients that come in and there’s very little information available (HC10).

One of the reasons that there is often limited information on both the health and social history and beliefs of immigrant or refugee clients is that there are no expectations that providers use the DSM-5 in their assessments. Care providers select their assessment process, and many are uncomfortable and unsure of what to ask immigrant and refugee patients. As one social worker noted,

A barrier is people’s fear of being curious around other people’s belief systems, right and not knowing how to ask those questions because of their own kind of discomfort with it…people are uncomfortable asking the questions or even asking people if they identify with a particular culture…What is your faith?…Just even getting to that comfortable level of asking that question (HC5).

This discomfort can prevent important issues being explored with clients, especially when they have a different linguistic, cultural, or religious background than the care provider.

Respondents noted that there was no mandated cross-cultural assessment tool across mental health services. Few are familiar with the section on cultural psychiatry in DSM-5 and the cultural formulation in the DSM-IV, and those who are, felt they needed to collect more detail for a robust transcultural assessment. There are gaps in the collection of linguistic or cultural information concerning patients from the emergency department: “There’s not even a little section to ask about do you think there are any cultural or ethnically relevant aspects to this patient that are worthy to note” (HC11). Such information in referrals could help providers prepare for the appointment by booking an interpreter or arranging for religious accommodation. The lack of culturally specific assessment tools was emphasized by a mental health clinician: “Our tool is what it is; there’s nothing built into that tool to allow for differences; cultural, the language or religious or dietary differences. Nothing” (FG1).

A reason there is no consistently used transcultural assessment tool in mental health was attributed to the difficulties in applying the DSM objectively across all situations. As one psychiatrist stated,

Even if you look at the evolution of DSM, it’s still a very – I mean, the first thing to remember is that psychiatric illnesses are real, but the ways we understand them are completely made up. It’s a bunch of people sitting around a table and these are the criteria to call it schizophrenia. It’s very arbitrary and it is very western based…We have to remember that unlike other areas of medicine that have more objective measures, in psychiatry diagnosis and symptomatology is very arbitrary and it’s a western construct (HC11).

Some providers include cultural and language assessments, but in general, these are used inconsistently and lack comprehensiveness. The Montreal Cognitive Assessment (MoCA) is a brief cognitive screening tool available in many languages; however, it lacks a transcultural approach. The Breslau screening tool that uses seven questions to assess PTSD was also mentioned, but it was not widely used (HC10). The O-CAN (Ontario Common Assessment of Need) was seen as too simplistic: “the last question is: is cultural issues a part of your life, and the answer is yes or no…And the previous question is: is religion an integral part of your life, yes or no?” (FG1). Participants noted that these yes or no questions from the O-CAN would not elicit the information needed to inform a culturally appropriate response. Further, it was noted that the appropriate timing of questions should be taken into consideration. One respondent noted,

the client’s past may be such a traumatic experience and they may be so psychotic at the time, that you’re not really going to get maybe the correct or accurate information because it may be tied up with their psychosis. And it may be so traumatic sometimes that bringing that up right now is counterproductive. (FG1).

If patients are in crisis when they meet the provider, questions about trauma may not be productive until the patient is more stable or a relationship of trust has been developed with the care provider.

The lack of standardized assessments for transcultural mental health leads care providers to adapt practices on an ad hoc basis. For some this meant doing a shorter screening at triage, a brief cognitive assessment (with an interpreter if needed), and determining the religious requirements for either a male or female health care worker (HL1). One respondent noted the need to tailor the assessment based on cues from the family, such as delaying questions around sexuality until they had established trust (HL2). The complexity of assessments with an interpreter also requires adaptation and adds the potential for misunderstanding. Many respondents spoke of the importance of training for working with interpreters, although this training was not available to them. As one program manager in mental health care explained,

So you know, in the emerg [Emergency Department], where I think they use them [interpreters] quite frequently, you learn by doing and you learn from a more experienced nurse or whatever. I think it should fan out across the organization where it might be used less. It’s probably more challenging. There wouldn’t be any formal training that I’m aware of, no. (HL4)

The level of skills of the care provider and the interpreter, as well as how they work together can affect the entire assessment process. Despite an overall dearth in training and formal processes for transcultural assessments, decision-makers and providers rely on their own knowledge, skills, and intuition, with many emphasizing a generic patient-centered care approach.

### The desire for a narrative approach

All respondents highlighted the need for a more encompassing approach to transcultural assessments. One social worker stated the need for “acquiring kind of thick descriptions of people’s lives, thick understanding of what’s going [on] – what their history is” (HC5). Knowing the patient story, such as past traumatic experiences, was necessary to support effective care, as the provider would better understand the basis of the clients’ mental health state. Provider wanted details not only about their past, but also about what they are currently dealing with, such as a family break-up or resettlement difficulties. As a physician leader in the emergency department explained,

To start with there’s usually a complex history, because most people who have an easy history don’t have many mental health issues, some do, but most don’t, right. So there are some complex issues … Then if you layer on top of that the fact that they’re…not English speaking as a primary language and you layer on that the fact that they’re immigrant and then you layer on the fact that…they’re refugees, the complexity of that piece of pie is really hard and you know…there’s gonna be lots of things potentially missed (HL3).

A nurse case manager noted that through exploring personal histories of immigrants or refugees accessing mental health services:

We get a really thorough psycho-social history on the person. It’s really, really important that we know where is that person from, what were the events of their life like. In particular, it’s important to focus [on the ages] between…13 and 30; those are really significant, those are our strongest memories. And if that person lived through a war during that time that can be significant. Or maybe they had really good things happening during that time and we need to tap into that. But getting to know who the person is, is fundamental in our care plans (HL1).

As one health care leader described “my approach to assessment is always I want to have it as a narrative. Like, so put together a story… if they’re somebody from an immigrant or refugee population, obviously, that’s going to be a big part of the story, right?” (HL7). Care providers noted that collecting the story allows them to get to know their patients, their roots, their work experience, their cultural practices, their religious beliefs, and their migration journey, including any potential traumatic experiences (HC10, HC8, HL7). Knowing this type of information would allow for a patient-led approach for the care plan, tailored to each client.

An occupational therapist emphasized the value in asking them how they view health care, as “there is such a difference from culture to culture even in terms of how they view it [the role of health care] and their needs” (HC10). Others noted the importance of “trying to get an idea of what things were like in the life that they came from” (HL7) and are asking immigrant families “what do you imagine your life looking like here?” (HC8). Respondents noted the importance of getting background information by “doing your homework…to understand the culture” (HC10). Participants emphasized the value of getting information from the patient’s support network, but reinforced that the focus should be on the patient’s current experience “to allow for their own description of what it is that’s happening to them” (HC5).

Ensuring a comprehensive patient narrative is obtained can be a lengthy process: “you’re looking for explanations in the current context, which has multiple new variables…the transition context, and then the pre-transition. And that’s then mapping on pre-morbid, morbid and current mental health status…No wonder it takes nine hours” (HC9). An in-depth history helps care providers understand a patient’s strengths and challenges, but it can be triggering. The patient may be suppressing difficult memories, “because sometimes again they want to hide that in a sense right, because it’s something that you know it’s personal to them and can cause memories to occur…But I think it’s important to understand” (HL3). In addition, health care providers noted the lack of time as a barrier to a full assessment.

Other care providers highlighted that dealing with historical traumas often has to be postponed in order to focus on immediate needs and goals. “It may be around safety issues, housing, making sure their children are safe and have food, and all those basics. With any of my clients, if those basic needs aren’t met, you’re not going to be able to do in-depth other work treatment with them” (HC10). Although many immigrants and refugees may have deep traumas, they also have tangible immediate challenges in their adjustment to life in Canada, navigating housing, school for their children, not knowing where to get information they need to establish themselves (HC14). One emergency department physician was clear that the assessments for immigrants and refugees, especially those with limited English skills, needs to first focus on those basics:

To run through a screening checklist: Who’s at home? What are the resources available to you? Who’s working? Who’s not working? Are there kids? Did you eat today? What did you eat today? How are you eating? And all of those things as a screening tool – for me, screening patients is sort of the first thing. Once I screen them, you very quickly know who you need work on (HC17).

### Appreciating cultural differences

Multiple providers emphasized that understandings of mental health are culturally embedded, and that the way we label psychological disorders is culturally biased (HC11, HC12). As one social worker explained: “with some cultures it would be easy to for instance suggest that this person perhaps is experiencing some form of psychosis, when in fact culturally that may be very acceptable to have that particular thinking or thought process” (HC3). Another social worker also echoed this perspective:

I’m trying to be aware that there could be a very different understanding of the situation than what I’m having and trying to really open the door and allow for that understanding, to come out, right?…I might say they’re having some auditory hallucinations, they’re whatever. But to them, ‘I’m hearing the spirits of my ancestors speak to me.’ You know what I mean? So is that a negative thing to them? (HC5).

An additional respondent noted:

Someone who is psychotic from one background might look quite different than someone who is psychotic from a different background. So if something doesn’t quite make sense and they don’t look quite the way we expected them to, to remind yourself that maybe their particular unique background might be contributing to why they look this particular way (HC11).

The assessment process itself can be a tool to explore cultural interpretations of mental health. As one psychologist shared,

The assessment really is more on trying to get their understanding of those issues because different cultures have different understandings of mental health issues. Trying to get those to be appropriate...to bring in as much cultural relevance of that therapy model in order to try and make it work (HC 12).

A psychiatrist reflected: “over the years I’ve become familiar with certain issues that tend to crop up in certain cultures and certain patterns tend to repeat. So it’s more that I just remind myself of that and if I see something that doesn’t quite fit, I see if I can make sense of it looking at it through that lens” (HC 11). This perspective resonated in the comments from a social worker:

I don’t think there’s anything wrong with allowing some of that knowledge to influence some of the questions that you’re asking, right? I…have learned about, you know, culture in Iran…that might cue me to ask certain questions, right? So I don’t think it’s a bad thing to ask to have some of that knowledge or a little bit of understanding (HC5).

Acknowledging cultural difference is an important beginning for effective transcultural care, yet it is important not to make assumptions based on these cultural differences. Instead, respondents recommend open-ended questions that allow the patient to lead the discussion results in more accurate assessments (HC2). The level of acculturation to Canadian society was a key factor in the ability to communicate and articulate health needs (HL3, HC2). A health care leader shared the story of an immigrant girl who initially presented for belly pain, but only years later could explain that she was being sexually abused:

She didn’t have the tools, the skill or the age ‘cause she was an immigrant, she spoke very little English, was depending on an interpreter to tell me her issues, versus now where she’s got six years of education, more, you know, more Canadianized for lack of a better word, right? And a little bit more empowered and now could bring the issue forward (HL3).

That somatic presentation was noted by some respondents as more common in women and girls in some cultural groups (HC9).

Understanding the importance of cultural factors in experience and communication of health concerns should not lead to assumptions about its relevance for everyone from that cultural group. As a social worker stated, cultural education is important, but interactions should be focused on exploration:

Just how to ask questions, not specifically that you have to be aware of every cultural process. Because I find that I get to know a lot about my patients, about asking them what their interpretation of their culture is. Because, again, it means different things to different people. So it’s not that I feel I have to be well versed in everything. And I admit fully when there’s something I don’t know, ‘I have no idea about that,’ ‘Can you tell me what that is?’ ‘How does your family respond to it?’ (HC15).

Problems can arise when care providers bring culturally based assumptions to the assessment, “maybe not necessarily in a negative way, and not meant to be malicious but some of those ideas, that people have learned a little bit about a culture and come in with some assumptions” (HC5). The best way to avoid misunderstandings is to take a patient-led approach to interpreting cultural meaning, allowing patients and families to guide the role of culture in their healing process.

### Family-based approaches

Family-based care is a valuable approach for mental health treatments with immigrant and refugee patients. Understanding the experiences that families have been through is an important beginning for the mental health assessment. Pre-migration experiences shape family norms and thus need to be understood by care providers. Community service sector and resettlement agencies were identified as facilitators of understanding the pre-migration experience:

I found it very helpful to have those other representatives from the community there that were aware of the story and also kind of aware of mental health concerns in that population. So they could say, ‘Okay, well you know when this family came this was going on.’ And that was very helpful for us to know;…they could give us some more kind of narrative around the experience with those family members (HC6).

A social worker noted the need to be aware of family perceptions about mental health to effectively engage in patient care:

I might be looking for certain cues or flags that tell me what’s not working, right? Like what, you know, what are some of the family dynamics that are going on here that might be influencing the situation, being very curious about how people are understanding this themselves,…how are they understanding themselves and this experience. How’s the family experiencing this? Because I may look at a person and think, oh this looks xyz to me, but the family might be sitting beside me going ‘my child has been taken over by a demon.’ (HC5).

As a psychiatrist explained, “I like to have families involved with the care of patients. I’m fairly comfortable having family meetings with people from different backgrounds, and I can tell too they’re comfortable having these meetings” (HC11).

This family-centered approach includes being able to understand how care plans and therapies impact the family. A social worker explained that it is possible to cause harm even when one has the best of intentions:

We thought we were pretty progressive when we bought the anatomically black dolly…But on a bigger scale, we had no sense of what that would be for a family that comes with a paper bag worth of their worldly goods, and then walks into this rich, toy, plastic, white, blonde, doll environment and she said to us, ‘now they’re going to – they’re going to ask me to buy this.’ And that wasn’t at all our intent. But the damage was done (HC9).

A social worker explained that sometimes the focus is on rebuilding family. A refugee woman was devastated and depressed when her husband left her not long after moving to Canada.

I did the assessment [of her] family of origin. We - she was able to reconnect with her cousin. And the cousin, who didn’t see her for 20 years, somehow was at the time of her life that she was able to help, and that made - I got a letter from her not long ago that made a big difference…It’s almost like rebuilding an environment (HC4).

Through this reconnection, the patient was able to strengthen her wellness plan and shifted her focus to one of hope for the future.

Other providers noted that many immigrant and refugee families experience trauma that is collective, layered, and intergenerational. These realities benefit from a family-based approach that recognizes “it’s not just the child’s trauma. It’s the child’s trauma plus the child’s experiences of the mom’s trauma. And then what - how they got here. And what has happened here” (HC9). The family-based focus for mental health was seen as crucial to both understanding the context of the patient’s life and developing a plan to rebuild the family unit to process their collective trauma and facilitate resilience.

### Challenges and complexity of trauma assessments

The experience of trauma is a significant challenge in many immigrant and refugee families. A social worker spoke to this challenge, noting that families experience trauma before, during, and after migration. Health care staff may recognize

some of the difficulties and adjustment to culture. But not necessarily the specifics of how they are going about integrating into the [local] community and finding connections here. And so I think we often go to those, you know, if we were to look at everything through a trauma lens we often look for the big T traumas but we don’t always look at the small t traumas, right? And there are all sorts of, like, little difficulties when it comes to kind of integrating into the community that I think we’re not necessarily aware of (HC6).

An emergency department physician noted that sometimes trauma goes unrecognized, sharing that a patient “had already been screened by a student who hadn’t picked up any of her previous trauma” (HC17).

Care providers spoke about the need to support patients to process their trauma, but to approach this with caution:

There’s some danger in that too, in a setting like an emergency department, right. If somebody’s presenting and they’ve, you know experienced something really horrible in their life, is the emerge [Emergency Department] the appropriate place to open all of that up with them? Not necessarily, right. So, to try and get an understanding of what some of that history is in a way that’s safe for them because if we’re not keeping them, then we’re sending them back out into the world and we just opened up the Pandora’s box of things that have happened to them in their life, right? So trying to understand, get some understanding of their experience. As well, also doing it in a way that’s safe for them and isn’t going to put them at greater risk than they may have presented with, right? (HC5).

Providing refugee patients appropriate opportunities to tell their trauma story was seen as key to the healing process, as long as it is approached carefully. As one psychiatrist explained:

We sat down with her and just part of my routine screening is to open the door, and say ‘sometimes people experience trauma. If you’ve experienced trauma and want to talk about it, I’m here.’ Just open the door, very, very non-confrontational and she just sort of poured her heart out. And I think the term I usually give it is life retelling, I think that’s the formal term for it. And told me about how she was a young girl and witnessed – she was quite young at the time, like 17 or 18 – and she’d witnessed her father and mother slaughtered in front of her…I was definitely the first doctor she had seen in a very long time, if not ever. And I don’t know that anybody had actually opened that door for her, I don’t know. I don’t think her trauma ended, possibly, until that day. Like the actual trauma, I think maybe that day was the first day of her healing (HC17).

Care providers need to be able to share clinically relevant information from these patient trauma stories with the care team, so the patient does not need to keep retelling their traumatic history. In this coordinated approach, the doctor deferred trauma screening to other members of the team, “and then feels very comfortable saying, ‘I’m not going to ask you to tell me the story, but I know something bad has happened to you and I’m going to ask you a bunch of other questions that relate to that without talking specifically about that’” (HC9).

The complexity of trauma among refugee populations demonstrates the value of an interdisciplinary team approach, as one psychiatrist asserted:

There isn’t one person that can - that can do it all. They’re – it’s complex. Usually the patients present with that profile usually have a traumatic background, it complicates things…We’ve constantly talked as a team, you know, do they really need to come and tell their story 50 times? Like, we – we need to find some way to streamline it, to just get the treatment started instead of, you know, seeing different – different people. So I really see, you know, a specific…trauma team that has the cultural sensitivity, but also has the interdisciplinary part. (HC13)

Through recognizing these complex challenges, respondents identified the need for a trauma-informed coordinated team-based model of care.

### Recommendations from respondents

Overall, respondents found assessments with immigrant and refugee populations challenging for multiple reasons, such as the need to work with interpreters, different perceptions of mental health, complex trauma, and the lack of time for completing the interview, which takes longer in these situations. In addition to through adopting a flexible, family-centered approach where assessments included rich descriptions, participants highlighted the need for a standardized mental health assessment process for immigrant and refugee patients in mental health. They also noted that assessments could only be effective if an interpreter is available. Respondents from a focus group asserted: “if we did have some systems in place to assess people better and to get a better understanding of where their needs lie and what the huge cultural differences are through a basic screening process with an interpreter, that would really help” (FG1). The respondents emphasized the need for a process or tool that integrates the interpreter and ensures that care providers are trained in using interpreters.

A consistent approach would reduce unnecessary variation and ensure that important things do not get missed, either due to care providers’ uncertainty, discomfort, or lack of knowledge. The assessment tool could allow staff autonomy and judgment, while providing support for the specific challenges of immigrants and refugees. Standardizing assessments would help staff who might not have the training and experience (FG1), and overall would improve the quality of care for immigrant and refugee patients (HC2).

There was an equally strong consensus around the need to be flexible and adaptive. Care providers need to be attuned to potential trauma triggers, and to ensure that the patient feels ready and safe with the providers and the environment. As a nurse leader noted about hospital-based care:

This is a very busy area, a very active, you know, environment for them to come into. Whether there be may be a separate area that is, you know, less stimulus, more isolated…They feel that if they are going to come into hospital to be assessed that they would feel more comfortable without the loud overheads going off and [security]…doing their rounds through there randomly and setting off a lot of the PTSD stuff (HL1).

Another important recommendation focused on building the knowledge and capacity of health care staff and organizations. Improving provider knowledge and organizational capacity also emphasized the value of training in trauma and transcultural informed models of care, interpretation use, and practical forms of interactive education (HC8, HC6, HC2).

A mental health care leader emphasized the importance of a flexible patient-centered approach for improving assessment processes for transcultural, trauma-informed care with immigrants and refugees:

I guess it’s really about how do we individualise the care plan to this individual and say okay, we’re all focused on healing this person, how are we going to make this work? Because we don’t want staff to say cookie cutter, oh, this worked for that last patient from Somalia, so it’s going to work for this patient, it’s going to work for this patient. Like it’s not going to work that way…I know our community partners have said, you know, this is about learning the questions to ask, not just making assumptions, as you say, but having an awareness that there are ways to get at that information… I call it being culturally curious, you know, not being afraid to ask. Not putting your foot in your mouth either, but you know, being curious enough just to ask a respectful question about how is the best way for me to work with you?...I see it as just being patient-centred (HL8).

## Discussion

Our study has demonstrated many complex issues to consider in the assessment of mental health disorders in immigrant and refugee families. Although current practices are inconsistent, respondents emphasized the need for a thorough yet flexible and adaptive approach that explores differences in cultural interpretations of mental health. The DSM-IV and DSM-5 provide guidance for practitioners, but their focus on diagnoses, while important for psychiatry, may not provide relevant information for providers in other areas of mental health who do not diagnose, nor have the time for the cultural formulation assessment. Questions within the DSM documents can provide valuable guidance, but it does not fit all clinical settings or needs. The DSM-IV and DSM-5 could be used to guide the creation of unit-specific assessments that fit the goals of those clinicians and clients. As Alacrón has argued, “very few academic or training centers, mainly in Canada, the U.S. and Europe, faced up to the tasks of exploring the feasibility, usefulness and practical applicability” of the outline for a cultural formulation of the DSM-IV [23]. To ensure a high level of competency and responsiveness to cultural difference, health care providers need supports and tools to increase their comfort in having these difficult conversations [15, 24]. This includes training in the use of the cultural formulation, as well as time allotted to carry it [10].

Care providers seem unaware of the materials they can access to increase their skills in transcultural care. As argued by Hebebrand et al., “practitioners may enhance their cultural competence through relevant knowledge accessible through refugee-specific websites” [25]. In order to ensure care providers do this preparatory work, organizations should provide continuing professional development opportunities in transcultural skills. It is possible to improve the comfort of health care workers to engage in difficult conversations in a culturally competent and responsive manner, through education and resources. Unfortunately, research has shown that staff working in a hospital setting are often not able to get professional development time to participate in such training [12]. Ferrari et al. note that patients also need to be provided with concrete recommendations and resources to support their mental health [13].

Improved access to and use of transcultural assessment tools would be valuable in the context of immigrant and refugee mental health care. Understanding the issues that shape their mental health could improve the appropriateness of the approaches used. Knowing the potentials of re-triggering trauma would ensure that care providers are more sensitive to these issues when taking a narrative history. Indeed, research on cultural formulation by Kirmayer et al. note “the prevalence of dissociative and somatoform symptoms, leading to misdiagnoses of psychosis, personality disorder, or malingering” [26]. Transcultural trauma assessments are more time consuming and need to be supported organizationally. As Ferrari et al. have noted, “the lack of time to properly discuss mental health problems with clients” is a barrier in the health care system [13].

Another benefit of transcultural training and tools is to develop understandings of how mental health can be informed by cultural values. As Adeponle et al. demonstrate, “ethnic differences in psychiatric diagnosis may arise because clinicians attribute and interpret symptoms differently” [16]. Such assumptions can lead to misdiagnoses, especially in mental health, leading Sundvall et al. to assert “the need for the assessing clinician to have a broad understanding of variations of risk factors and to avoid the stereotyped view” [14]. Each client will interpret their mental health framed through their own unique perspectives; allowing them to explain their experiences can prevent culturally biased assumptions from providers. As Kirmayer et al. demonstrate, the consequences of cultural misunderstandings can be significant, including “incomplete assessments, incorrect diagnoses, inadequate or inappropriate treatment, and failed treatment alliances. These problems are costly, both in terms of increased service use and in terms of poor clinical outcomes” [26]. Choosing appropriate processes for exploring past trauma is also key in supporting effective assessments and care oriented to the refugee experience [27, 28].

Since the mental health problems of immigrant and refugee patients are often based in traumas affecting all family members, many studies emphasized the appropriateness for a family-based approach [1, 29–38]. Supporting the family and understanding their collective experience of mental health and trauma is a valuable approach in transcultural care [39]. Multiple studies echo our findings that successful treatment plans are most effective if families can play a supportive role in the healing process [30, 33, 34].

Weine et al.’s study demonstrates that rapidly changing roles, obligations, and connections of traumatized refugee families in the new country create additional coping challenges [35]. Solobodin and de Jong’s systematic review concluded that secondary trauma among immigrants and refugees was common, where “family members are affected not only by the changes experienced by the primary trauma victim but also by the victim’s transmission of trauma-related experiences, behaviors, emotions, and attitudes” [1]. Cultural norms of male dominance can be a barrier for women accessing the services they need, so attention needs to be paid to the relationship dynamics to ensure all members are safe from abuse within the family [11].

Our respondents noted that the resettlement process itself can be traumatic, a finding that is reflected in the literature [1, 21, 29, 40]. Families are facing a wide range of difficult pre- and post-migration experiences that create collective trauma [1, 33, 38, 41]. As Ehntholt and Yule note, “frequently refugees need to discuss practical or present difficulties rather than past experiences owing to the high levels of social and material adversity under which they are often living” [3]. Thus the mental health care response should ensure “balancing past trauma with present-day resettlement and acculturative stressors (e.g., housing, employment, health care)” [24]. This collective trauma adds significant complexity to mental health assessments and treatment plans. And since these traumas can be presented in unfamiliar or subtle ways, not all care providers are able to correctly assess them among immigrant and refugee patients [42].

Mental health care for immigrant and refugee patients and families is complex and thus needs an approach that is well informed and supported by an organizational commitment to standards of care. In addition, complex family-based trauma care, as recommended by Mendenhall and Berge, requires an interdisciplinary team-based approach [43]. The skills needed for this team include a wide range of transcultural practices, including training with the cultural formulation interview and working with interpreters [10, 26, 39]. Despite wanting flexibility, respondents emphasized the value of a standardized transcultural mental health assessment process, as seen in a range of studies [1, 2, 29, 44]. As Adeponle et al. note,

No gold standard method exists for diagnosis in cross-cultural settings. Although investigators have traditionally advocated for the use of structured interviews and strict diagnostic criteria as a way to reduce biases that may occur in clinical settings studies indicate that neither approach yields more accurate diagnosis or eliminates clinician bias [16].

This suggests that any assessment tool needs to support “flexibly tailored, multilevel interventions that are implemented in creative and engaging ways” [24].

## Conclusions

The respondents in our study demonstrated that ensuring mindful, reflexive practices that are transcultural and trauma-informed will go a long way in improving the health care experiences and healing journeys of immigrants and refugees in the mental health care system [24]. Although guidelines exist to inform such a practice in the DSM-5, some of the requirements, such as a supported learning environment that included education, resourcing, and standardized tools, are not in place across mental health services in London, Ontario. In addition, the complexity of intergenerational and family trauma highlights the need for using a family-based model of care, which is also poorly supported through existing models for appointments and billing. Families are collectively facing past and present challenges, ensuring the family as a whole is collectively embarking on their healing journey builds resilience to support that healing.

This strong endorsement from care providers and community agencies led to the development of a transcultural, trauma-informed family-based mental health consultation service. The next research phase will assess the benefits of this model for improved health outcomes of immigrant and refugee families experiencing trauma. Intervention research that focuses on outcomes is needed to demonstrate effectiveness of a family-based approach that explores social and cultural determinants of health of immigrants and refugees as they resettle into their new lives [11, 27, 44]. This study demonstrates that it is a step in the right direction.
